# Landslide Susceptibility Assessment Using an AutoML Framework

**DOI:** 10.3390/ijerph182010971

**Published:** 2021-10-19

**Authors:** Adrián G. Bruzón, Patricia Arrogante-Funes, Fátima Arrogante-Funes, Fidel Martín-González, Carlos J. Novillo, Rubén R. Fernández, René Vázquez-Jiménez, Antonio Alarcón-Paredes, Gustavo A. Alonso-Silverio, Claudia A. Cantu-Ramirez, Rocío N. Ramos-Bernal

**Affiliations:** 1 Department of Chemical and Environmental Technology, ESCET, Rey Juan Carlos University, 28933 Móstoles, Spain; 2 Grupo de Investigación en Teledetección Ambiental, Unidad Docente de Geografía, Geología y Medio Ambiente, Área de Geografía, Universidad de Alcalá, Filosofía y Letras, 28801 Alcalá de Henares, Spain; fatima.arrogante@uah.es; 3 Área de Geología, ESCET, Universidad Rey Juan Carlos, 28933 Móstoles, Spain; fidel.martin@urjc.es; 4 Research Group on Technologies for Landscape Analysis and Diagnosis (TADAT), Department of Chemical and Environmental Technology, ESCET, Rey Juan Carlos University, 28933 Móstoles, Spain; carlos.novillo@urjc.es; 5 Data Science Laboratory, Rey Juan Carlos University, 28933 Móstoles, Spain; ruben.rodriguez@urjc.es; 6 Cuerpo Académico UAGro CA-93 Riesgos Naturales y Geotecnología, FI, Universidad Autónoma de Guerrero, Chilpancingo 39070, Mexico; rvazquez@uagro.mx (R.V.-J.); rnramos@uagro.mx (R.N.R.-B.); 7 Cuerpo Académico UAGro CA-178 Desarrollo Tecnológico Aplicado, Universidad Autónoma de Guerrero, Chilpancingo 39070, Mexico; aalarcon@uagro.mx (A.A.-P.); gsilverio@uagro.mx (G.A.A.-S.); 8 Ingeniería para la Innovación y Desarrollo Tecnológico, Unidad Académica de Ingeniería Dependiente, Universidad Autónoma de Guerrero, Chilpancingo 39070, Mexico; acantu@uagro.mx

**Keywords:** landslide, hazard assessment, susceptibility, automatic machine learning

## Abstract

The risks associated with landslides are increasing the personal losses and material damages in more and more areas of the world. These natural disasters are related to geological and extreme meteorological phenomena (e.g., earthquakes, hurricanes) occurring in regions that have already suffered similar previous natural catastrophes. Therefore, to effectively mitigate the landslide risks, new methodologies must better identify and understand all these landslide hazards through proper management. Within these methodologies, those based on assessing the landslide susceptibility increase the predictability of the areas where one of these disasters is most likely to occur. In the last years, much research has used machine learning algorithms to assess susceptibility using different sources of information, such as remote sensing data, spatial databases, or geological catalogues. This study presents the first attempt to develop a methodology based on an automatic machine learning (AutoML) framework. These frameworks are intended to facilitate the development of machine learning models, with the aim to enable researchers focus on data analysis. The area to test/validate this study is the center and southern region of Guerrero (Mexico), where we compare the performance of 16 machine learning algorithms. The best result achieved is the extra trees with an area under the curve (AUC) of 0.983. This methodology yields better results than other similar methods because using an AutoML framework allows to focus on the treatment of the data, to better understand input variables and to acquire greater knowledge about the processes involved in the landslides.

## 1. Introduction

Landslides involve 5% of natural disasters globally [1], which poses great risks as they are associated with other natural disasters, such as hurricanes, earthquakes or eruptions [2]. Besides, in recent times this natural risk has increased on a global scale due to urban development in areas prone to landslides, deforestation and increased regional or local rainfall caused by climate change [3,4]. For these reasons, landslides have negative consequences on the environment, material goods and human lives [5].

To reduce and mitigate the risk associated with this natural phenomenon, the processes of identifying and understanding the causes of landslides must be improved to promote prevention policies, early warning systems and recovery programs [6]. We define risk as the combination of the physics probability of an event happening (hazard) and the potential damage that this event might generate (vulnerability) [7]. For this reason, we must address the evaluation of the landslide risk into a framework, combining danger and vulnerability.

Regarding landslide hazard assessment, one of the most effective tools are susceptibility maps [8], which are understood as the spatial distribution of the probability of occurrence of a landslide [9]. These maps allow us to exploit the spatial relationship between the conditioning and triggers factors and their occurrences, thereby identifying areas where future events can occur [10]. Furthermore, landslides are strongly associated with topographic, geologic, meteorological and environmental factors [11,12]. Therefore, data sources containing spatial information related to these factors can determine the susceptibility of an area to these natural disasters [13]. In recent years, the use of methodologies based on geographic information systems (GIS) has intensified landslide susceptibility analysis. However, to choose the most accurate assessment of landslide susceptibility in a study area, comparing and testing different models is essential [14,15].

Methods used to develop susceptibility maps can be divided into three types: heuristic, classical statistics and machine learning methods [16]. Firstly, heuristic methods are based on the development of susceptibility index starting from landslide inventories, classifying the conditional factors according to a hierarchy [17], which introduces a subjective appreciation of the importance of each factor [18]. Secondly, methods based on classical statistics analyze the linear correlation between landslide and their conditional factors [19]. Into this category, we find models based on the value of information [20], the weight of evidence (WOE) [21] or the general linear models (GLM) [22]. Finally, machine learning methods utilize linear and non-linear relationships between landslides and the conditioning factors [23]. The latter are increasingly used to produce susceptibility maps due to the good results in environments where conditions are dynamic and complex processes [24].

Among the machine learning models used to calculate susceptibility, we find logistic regression, support vector machines (SVM), decision trees, k nearest neighbors (KNN), neural networks [25], Bayesian network [26], naive bayes [27] or fuzzy logic [28]. Although in recent times, research has also been focused on the use of assembler techniques, such as bagging, dagging, boosting [29], the use of deep neural networks (deep learning) [30] or the application of hybrid computational intelligence models [10,31]. Currently, studies focus on: (i) hybrid applications of various algorithms [5,10,21,31] (ii) comparison between algorithms belonging to different typologies [29,32,33], (iii) comparison of different models based on a single algorithm typology such as SVM [13,34], random forest [22,27,35,36,37] or neural network techniques [30,38,39], (iv) demonstrate the sensitivity of the models to how the data are sampled, how the hyperparameters of the models are configured or how the information is parameterized [40,41,42].

Due to the proliferation of different techniques, the burden of research is falling on the generation of models and therefore risk assessment lost sight of. Thus, the new automatic machine learning frameworks (AutoML), which facilitate models’ development, adjustment and evaluation, allow analysts, developers and scientists to focus on reflection, discussion and analysis of the results [43]. AutoML frameworks based on open-source libraries, such as Scikit-learn, bridge the different machine learning models design levels, boosting the data science process [44].

The main objective of this study is to assess the danger of a landslide, based on the development of a susceptibility measurement methodology, focus on the understanding of the data and deep knowledge about the causes and characteristics of the landslide, using for modelling an AutoML framework, comparing the performance of 16 machine learning algorithms. This automatic comparison of models places the burden of research on the causes of landslides and on the study of their conditioning variables, which means that there are better susceptibility predictions.

## 2. Materials and Methods

### 2.1. Materials

#### 2.1.1. Study Area

Guerrero is one of the Mexican federative entities. It is located in the southern region of the Mexican Republic, forming part of the South Pacific region [45]. The state of Guerrero is crossed from northwest to the southeast by the Sierra Madre del Sur [46]. In this state is found the tectonostratigraphic complex of Xolapa and Guerrero, which are in tectonic contact. The first presents a composition of metamorphic rocks and the second a sequence of metavolcanic rocks and slates [47]. Finally, this state is located on the Guerrero-Morelos old marine platform, consisting of a series of extensive limestone outcrops [48]. This state is frequently affected by hurricanes from the Pacific Ocean (the most common) and the Atlantic Ocean [49]. For example, in September of 2013, the area was affected by a serious stormy episode due to the conjunction of hurricanes Ingrid (formed in the Gulf of Mexico) and Manuel (from the Pacific), which caused floods and landslides [49].

#### 2.1.2. Landslide Inventory

The landslide inventory makes digitizing a Google Earth image obtained on 12 August 2014. Three different photo interpreters do the work. This inventory has 518 identified landslides, whose areas range from 21 square meters to 1.14 square kilometers, with 10,672 square meters being the average area. The precipitation caused by the hurricanes is the main trigger of the landslides, mostly earth slides type based on Cruden and Varnes classification [50,51].

This inventory has allowed us to develop the variable to be explained, the presence or absence of landslides. Rasterization of the landslide polygons performs to achieve this, based on the Landsat 8 resolution (30 m), obtaining a sample of 13,610 landslide points, which are the records of the dataset. On the other hand, we developed a random subset where we do not identify landslides. Join these two slides is used as a train-test dataset of 26,021 records. Regarding the generation phase of the susceptibility map, the total area was used, which represented a demonstration set.

#### 2.1.3. Data Sources and Explanatory Variables

Table 1 summarizes the variables used as conditional factors when predicting the presence or absence of landslides. Thirteen variables were selected based on the variables most used in the literature to develop susceptibility evaluations of landslides [21,33,52,53,54]. Based on these studies analyzed in previous works [52,55], these variables evaluated their effect on the generation of landslides. Selecting the variables for this study following two conditions, the availability of the information and the a priori effect these variables have on landslides.

**Figure 1:**
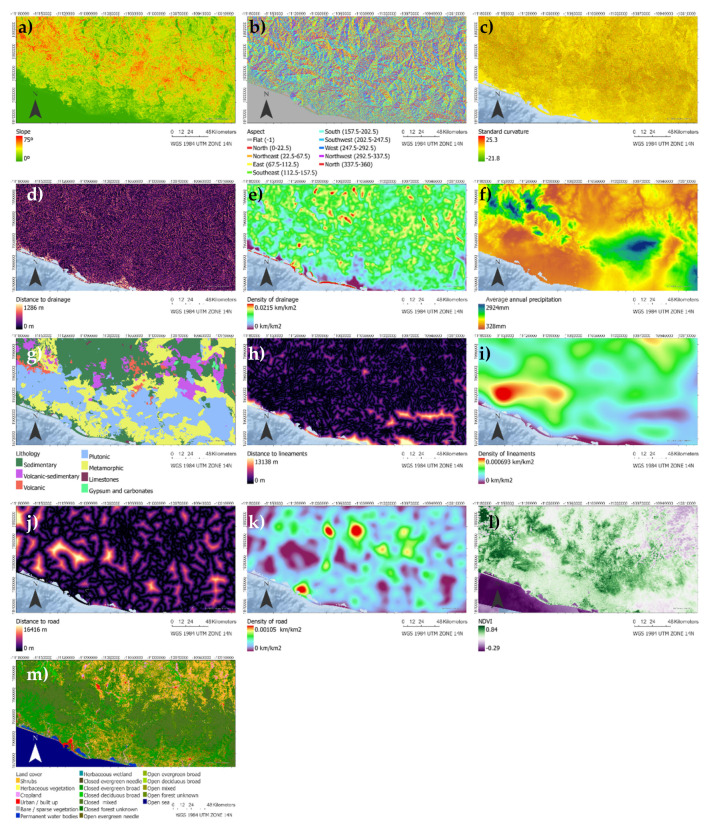
Graphical representation of the explanatory variables. Slope (**a**), aspect (**b**), standard curvature (**c**), distance to drainage network (**d**), density of drainage network (**e**), average annual precipitation (**f**), lithology (**g**), distance to lineaments (**h**), lineament density (**i**), distance to road infrastructure (**j**), road infrastructure density (**k**), NDVI (**l**), land cover (**m**).

Conditional factors constitute the passive elements that depend on the local characteristics of the landslide [56]. These factors correspond with the mechanisms within the landslide produce a reduction in the resistance to breakage [57]. The graphic representation of these variables appears in Figure 1. We used ArcGIS Pro 2.8 software tools to calculate distances and densities, Euclidean distance and Kernel density [58].

##### Shuttle Radar Topography Mission (SRTM)

SRTM is a near-global scale digital elevation model, which using radar interferometry. NASA JPL provides this product with a resolution of 1 arc-second (approximately 30 m) [59].

From this data source, we measured the slope, aspect, drainage network and standard curvature of the terrain, used ArcGIS software tools [60]. The aspect is categorized in accordance with the cardinal points, including a category for flat areas. This categorization performs according to Table 2.

##### Daily Surface Weather and Climatological Summaries (Daymet)

Daymet is a dataset with estimated daily meteorological parameters for North America, Hawaii, and Puerto Rico, with a resolution of 1 kilometer. Estimation algorithms and data processing are described in Thornton et al. [61]. This study used this dataset to measure the average annual precipitation between 1 January and 31 December of 2012, using a script in Google Earth Engine [62].

##### INEGI Geological Vector Data

Susceptibility assessment is very sensitive to geological variables, and the main spatial geological sources are the geological maps. Some studies use directedly (without further elaborations) the geological units from the geological maps, mainly based on the age of the rocks and sedimentation ages [63,64]. However, the geological maps are not elaborated for the specific purposes of landslide studies.

The geological information provided by the National Institute of Geography and Informatics Statistics of Mexico (INEGI) collects data about the origin, classification, and age of the rocks, including information about faults, fractures, volcanoes, mines, etc., at a scale of 1:250,000 [65]. This information was used to develop lithotechnical variable and those variables related to the lineaments. Geological information of the study area encompasses 47 different geological units. In this work, they were reclassified into broader units according to lithological criteria, genetic process (igneous and sedimentary), and among them, the geotechnical processes suffered (cohesion), which are potentially related to landslide susceptibility [22,40,47,66]. The best prediction is obtained when all the geological parameters are used together [40]. The provided multilevel information was reclassified and guided by expert decision. As a result, the geological formations were clustered into seven categories. Thus, sedimentary lithologies such as sands, silts, and conglomerates are the materials most susceptible to sliding. Conversely, the least susceptible materials are plutonic igneous rocks (granites, granodiorites, syenites), metamorphic lithologies (quartzites) and chemical sedimentary rocks (limestones and carbonates) (Table 3).

##### INEGI Roads Vector Data

Road infrastructure is related to landslides because of their destabilizing upper slopes through slope cutting, concentrating surface water, and hydro-logical patterns changes [67], overall in poorly constructed roads [68].

The cartography of roads, paths and elements associated with the communication network includes towns, places of interest and transport services, among others [69]. This cartography uses to develop the density and distance to the road infrastructure.

##### Landsat 8

The Landsat program, developed jointly by the United States National Aeronautics and Space Administration (NASA) and the United States Geological Survey (USGS), provides images of the Earth continuously since 1972, at a resolution of 30 m, including multispectral and thermal information [70].

Orthorectified scenes calculated in the upper part of the atmosphere (TOA) are developed from Landsat 8 Collection 1 Tier 1 images [71]. These images measure the normalized difference vegetation index (NDVI) from all scenes over eight days. In our study, the average of the scenes of the summer months (rainy season) of 2013 corresponding to the months with the highest photosynthetic activity.

##### Copernicus Global Land. Moderate Dynamic Land Cover

The global land cover service of the Copernicus initiative provides bio-geophysical information products to know the status and evolution of land cover on a global scale. These services include a global land cover product at 100 m resolution generated annually, using the PROBA-V satellite vegetation instrument. This product uses a three-level classification according to the land cover classification system (LCCS) class scheme [72].

### 2.2. Methods

**Figure 2:**
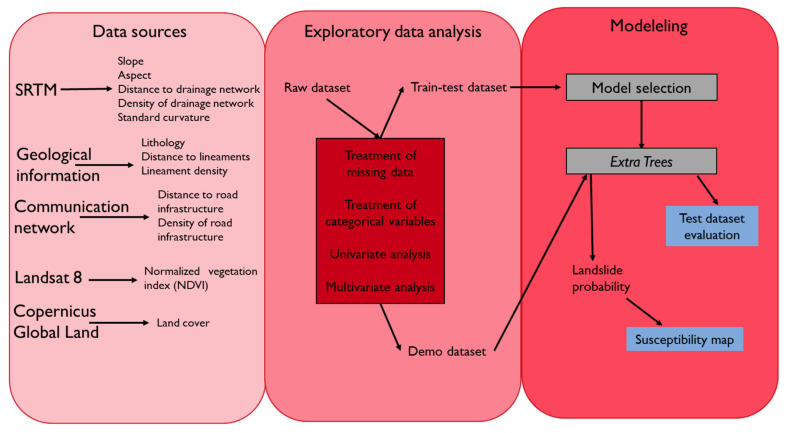
Workflow chart of the methodology perform to make the susceptibility map in this study.

Figure 2 includes the workflow followed in this study. The methodology used is divided into three phases. Firstly, we developed the variables for the study from the information sources to build the raw dataset. Secondly, we developed an exploratory data analysis, in which two datasets were generated, one for train and test and the other for make the susceptibility map. Finally, we made an automatic model selection based on the results of train and test data. With the best model identified, we develop a probabilistic prediction to make a landslide susceptibility map in the entire area of study.

#### 2.2.1. Exploratory Data Analysis

Exploratory data analysis identifies the structure of the data [73]. This analysis is divided into three parts: the study of the data structure, cleaning and filtering the data, and finally, a graphic study of the elements of interest [74]. In the first phase, we studied the size of the data, identifying if it is balanced, the types of the variables and finally, the missing data. In the second phase, an imputation and cleaning of the missing data and quantifying the categorical variables included in the data set are performed. On the one hand, in the last phase, perform a univariate analysis of each variable, including a graphical analysis of the distributions concerning the variable to be explained. On the other hand, conducted a multivariate analysis of Pearson’s correlation coefficient.

Concern about the imputation of missing data for average precipitation, an imputation by close neighbors (knn) was used because there is a spatial correlation between the different records [75]. Regarding lithology, we used an imputation based on the most frequent value of this variable. We only used record elimination on missing data for slope and aspect variables.

Related to the treatment of categorical variables are not ordered, we used a target encoder. This encoder replaces categories with a combination of probability as a function of the variable to be explained, based on the Bayesian empirical framework [76].

Finally, we made a standardization (z-score) of the dataset in algorithms not based on decision trees. This standardization minimizes the bias of those variables whose numerical contribution is greater in the classes segregation pattern [77].

#### 2.2.2. Model Comparison

The open-source Pycaret library [78] use in the model generation phase to be able to make a comparison of different machine learning models. However, the great diversity of models and techniques used to perform susceptibility model studies [79,80,81,82] makes it difficult to know which model will have the best performance. Therefore, the autoML framework is utilized in the search spaces process. This process facilitates: (i) finding the model that obtains the best results from a dataset, (ii) the optimization process of the model, (iii) the adjustment of the hyperparameters of the selected model and (iv) the evaluation of the results with the test set [43]. Besides, we used a cross-validation methodology (k-folds) of ten subsets to ensure that the results were independent of the partition of train and test data [83].

The 16 models compared in this study were: da boost classifier, catboost classifier, decision tree classifier, extra trees classifier, extreme gradient boosting, gradient boosting classifier, k neighbors classifier, light gradient boosting machine, linear discriminant analysis, logistic regression, MLP classifier, naive Bayes, quadratic discriminant analysis, random forest classifier, ridge classifier, SVM—radial kernel.

#### 2.2.3. Extra Trees Classifier

The extra trees algorithm was proposed by Gurts et al. [84] as a new tree-based assembly method to solve supervised classification and regression problems. This algorithm consists of applying strong randomization of both the attributes and selecting the cut-off point to divide the nodes of each tree. This algorithm consists of using strong randomization of the attributes and selecting the cut-off point to separate the nodes of each tree [84].

The model trained and evaluated with the train-test dataset has subsequently been used with the demonstration dataset to develop a probabilistic prediction of the entire study area, used to generate a landslide susceptibility map. The susceptibility is categorized into five levels (from areas with a very low probability of landslides to areas with a very high probability of landslides) used the natural cuts method (Jenks) [15].

## 3. Results

### 3.1. Exploratory Data Analysis

#### 3.1.1. Treatment of Missing Data

The train-test dataset contains 115 missing data distributed in the variables of average annual precipitation (37), lithology (70), aspect and slope (8). These missing data represent 0.44% of the total data. Therefore, these 115 records could be removed without affecting the dataset’s structure. Still, for maintenance, the higher number of registers in the dataset includes an imputation by close neighbors (knn) for annual precipitation and most frequency value imputer for lithology missing data. Therefore, we only used record elimination on missing data for slope and aspect variables.

We found 555,428 missing data regarding the demo dataset, which represented 1.35% of the total records. The same treatments performed in the train-test dataset to maintain consistency using the knn imputation for average annual precipitation and most frequency value for lithology. Thus, removing the rest of the missing data, which represents 0.07% of the records.

#### 3.1.2. Treatment of Categorical Variables

The dataset contains three categorical variables, aspect, lithology, and land cover. The aspect is divided into 9 categories, the lithology is divided into 7 lithological groups, and the land cover is divided into 17 covers. Concerning the treatment of these variables. In the case of lithology, whose categorization was ordered, an ordinal coding is done. However, a coding based on the objective was used for aspect and land cover, whose categories do not have an ordinal sense.

#### 3.1.3. Univariate Analysis

**Figure 3:**
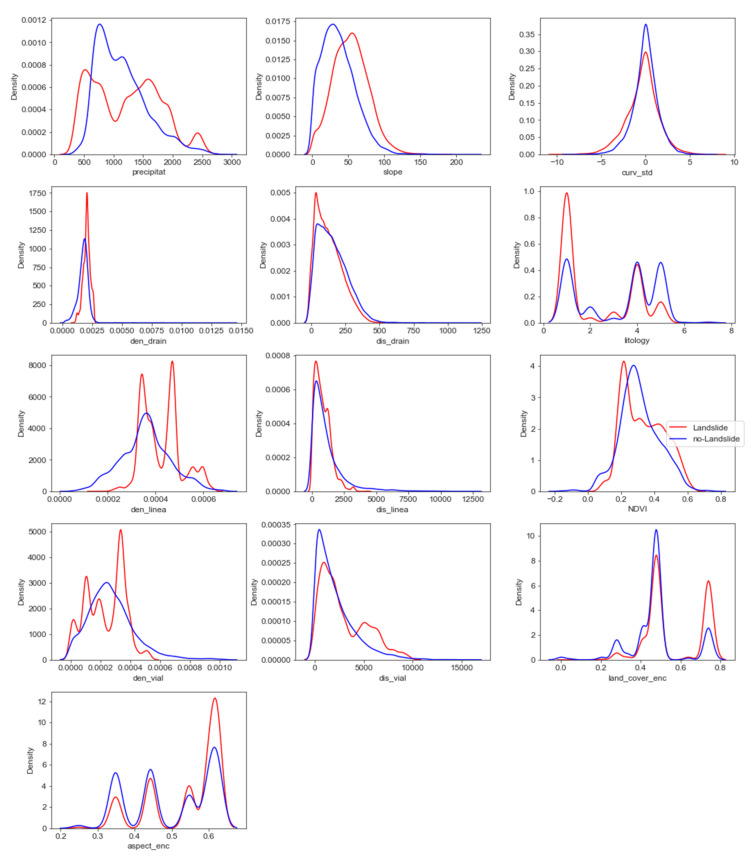
Graphs of distributions of the explanatory variables according to the variable to be explained (see text for explanation).

Figure 3 shows the different distributions of the variables depending on the variable to be explained. In addition, it is shown how some explanatory variables have other distributions depending on the variable to be explained. For example, we observe how average annual precipitation (precipitat) for the non-landslide class has a shifted distribution to the left, finding a maximum between 500 and 1000 millimeters. While in the case of the landslide class, two relative maximums recorded 500 and 1700 millimeters, which gives us an idea that there are two different patterns between the probability of landslide and the average annual precipitation.

Regarding lithology (lithology), in category 1, those areas with sedimentary materials have a differentiated maximum between the landslide and non-landslide classes. On the other hand, differences were observed in class 5, areas with metamorphic materials, contrary to those surveyed in category 1.

Concern to the density of lineaments (den_line), it is shown for the category of non-landslides that it has a distribution like the normal. In contrast, an irregular distribution is observed in the case of the landslides class. The density of road infrastructure (den_vial) describes in the variable density of lineaments observed.

Finally, for the rest of the explanatory variables, minor differences were observed between the two categories of the variable to be explained.

#### 3.1.4. Multivariate Analysis

**Figure 4:**
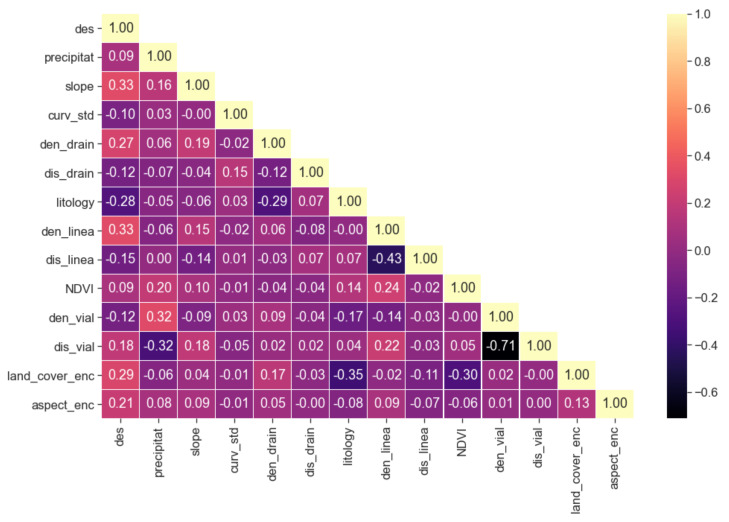
Pearson’s correlation plot includes explanatory variables and the variable to be explained.

Figure 4 includes the pairwise correlation study (Pearson) results of the different variables of the train-test dataset. In general, there are no high correlations between the variables.

The variable to be explained (des) did not observe a striking correlation with any explanatory variables. Instead, the highest correlations were observed between the distance (dis_vial) and density (den_vial) of the road infrastructure (−0.71). In the same way, these correlation values are repeated between the distance (dis_line) and density (den_line) of the lineaments (−0.43). However, for the case of the distance (dis_drain) and density (den_drain) of the drainage network, this correlation did not appreciate. Finally, we propose to drop the distance to road infrastructure (dis_vial) due to the high correlation with the density of the road infrastructure (den_vial).

### 3.2. Model Comparison

Table 4 shows the results of the 16 models adjusted according to the train-test dataset. These results are ordered according to the performance obtained. Table 4 shows how the models that got the best results for the dataset were those based on decision trees, both those of the bagging class (random forest, extra trees) and those of the boosting class (extreme gradient, catboost).

Regarding the results of the main statistics (Table 4), it is not observed remarkable discrepancies between model values. The models were able to correctly discriminate where there is a landslide from where there is not. In general, the results of the Kappa statistic show that the results of the last seven models can have a large random effect because their Kappa values are far from the results obtained in the other statistics. Finally, in terms of computation time, it is seen that the models based on bagging are faster than those based on boosting.

**Figure 5:**
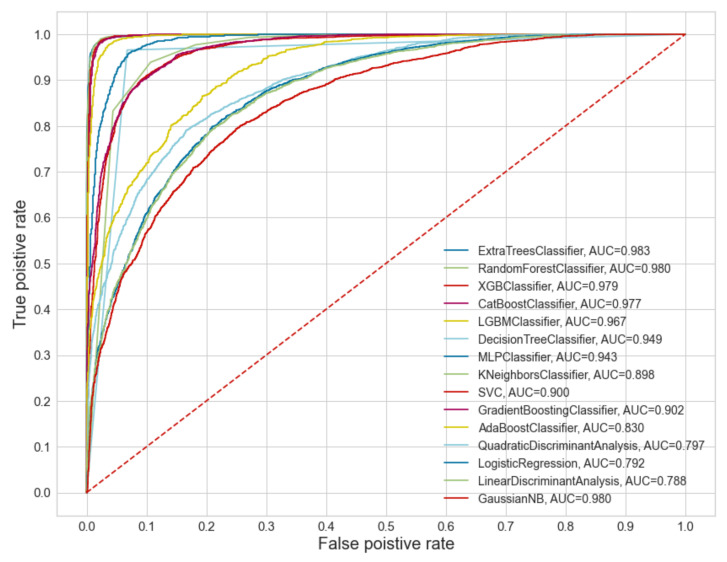
ROC curve of the comparison of the models used based on the test dataset. The dotted line corresponds to random results.

Figure 5 includes the ROC curve of the results, except for the Ridge classifier model, which does not provide information on the area under the curve (AUC) due to its characteristics. It is not observed great differences between the results obtained for the first four models (Figure 5). However, for these four models, we observe certain discrepancies in other statistics values.

On the one hand, it is observed how the two models based on bagging (extra trees and random forest) have very similar values in all statistics, except in computing time, in which the extra trees model is twice as fast as the model random forest. On the other hand, we observe that two models based on boosting (extreme gradient boosting and catboost classifier) have very similar statistics, except for the computation time. However, there are discrepancies between the statistics of the models based on bagging. These discrepancies are mainly observed in the recall and precision values. Firstly, it is observed how the models based on boosting have a higher recall than those based on bagging but have lower precision values. Secondly, the recall and precision values differences are higher in the boosting models than the bagging ones. These discrepancies can have consequences on the landslide predictions of these two different types of models. For these reasons, the extra trees classifier was selected as a model to be evaluated.

### 3.3. Extra Trees Classifier

**Figure 6:**
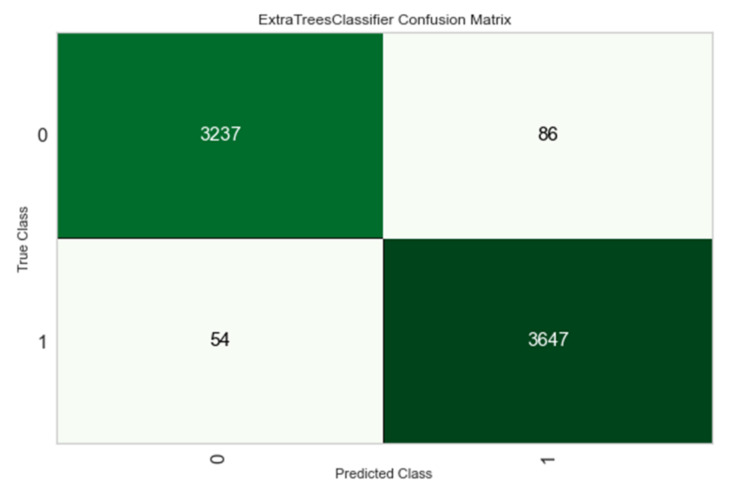
Confusion matrix of the extra trees model on the test dataset.

Figure 6 shows the confusion matrix of the extra trees model trained in the previous section (Section 3.2). It observed how the results were good for the four categories, underlining the high degree of success for the landslide category (1.1).

**Figure 7:**
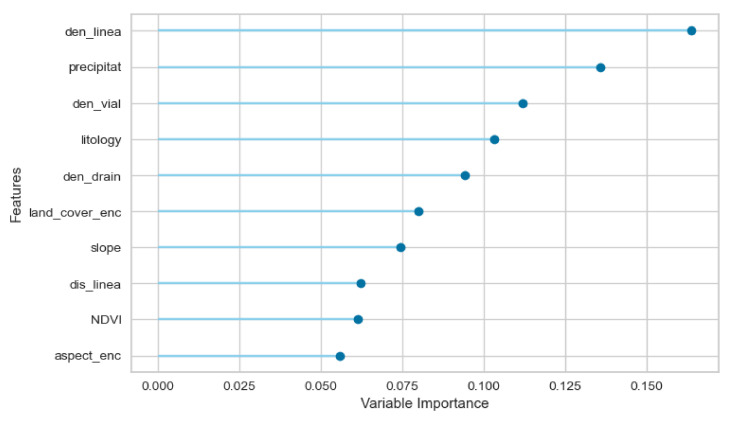
Importance of the explanatory variables, according to the extra trees trained model.

Related to the importance of the explanatory variables in the model (Figure 7), it is observed that four variables are over 10% of importance, two related to the geological characteristics, lineament density (den_line) and lithology (lithology), the average annual precipitation (precipitat) and the related to road infrastructure (den_vial). A second group can be identified, with the variables that have importance greater than 7.5%. In this group found a topographic variable, the density of the drainage network (den_drain) and one variable related to vegetation, the type of cover (land_cover_enc). Finally, a third group can be identified, with those variables of importance greater than 5%, in which we find a variable related to vegetation (the NDVI, two topographic variables (the slope, (slope), and the aspect (aspect_enc)) and a geological variable (the distance to lineaments, dis_line). The other two variables are of minor importance in the model. These are the distance to the drainage network (dis_drain) and the standard curvature (curv_std). In all cases, the kernel density variables are more important than their counterparts of Euclidean distances, observing large differences, as in the case of variables related to lineaments and drainage networks.

**Figure 8:**
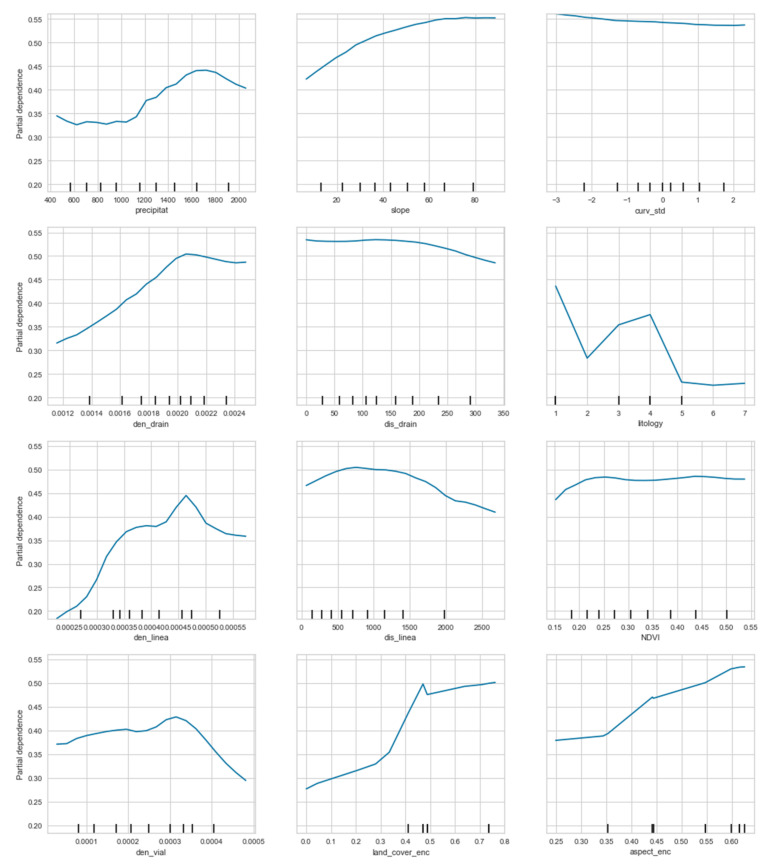
Partial dependencies between the explanatory variables and the variable to be explained (see text for explanation).

Figure 8 shows the partial dependence of the variable to be explained by the different values taken by explanatory variables. It is observed in cases where the partial dependence describes more linear curves, the less importance of these variables.

About lineament density (den_line), the curve describes an irregular rise up to a maximum close to the value 0.00045. The average annual precipitation (precipitat) observed how up to values somewhat greater than 1000 millimeters. The partial dependence describes a flat curve, with irregularities, relating a convex curve from that point, reaching its maximum at values close to 1700 millimeters.

Regarding the lithology variable (lithology), which is a categorical variable, the partial dependence is maximum in categories 1 and 4, results like those shown in Figure 3. In this case, it did not keep a relationship between the model and category 5, which seemed like it would give the model a lot of information based on the distribution chart.

### 3.4. Demonstration

**Figure 9:**
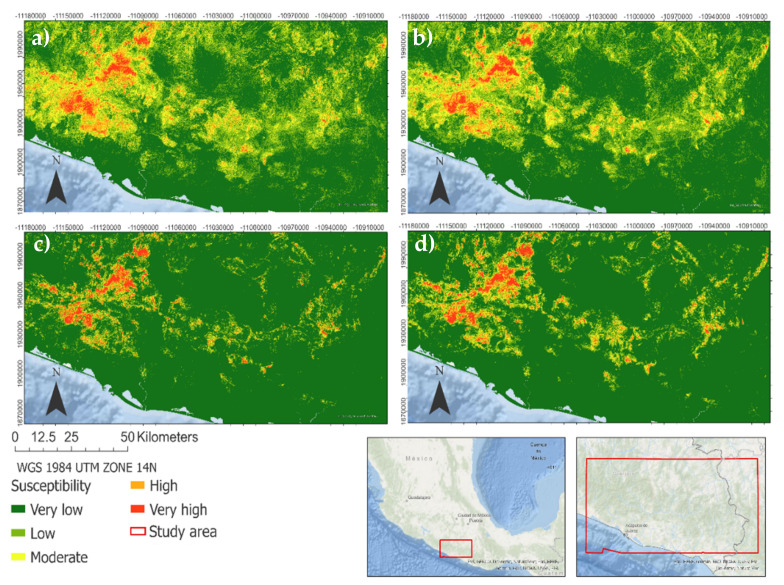
Landslide susceptibility map based on the probabilistic prediction of the (**a**) extra trees, (**b**) random forest, (**c**) extreme gradient boosting, (**d**) catboost classifier. At the bottom, the figures show the study area location.

**Figure 10:**
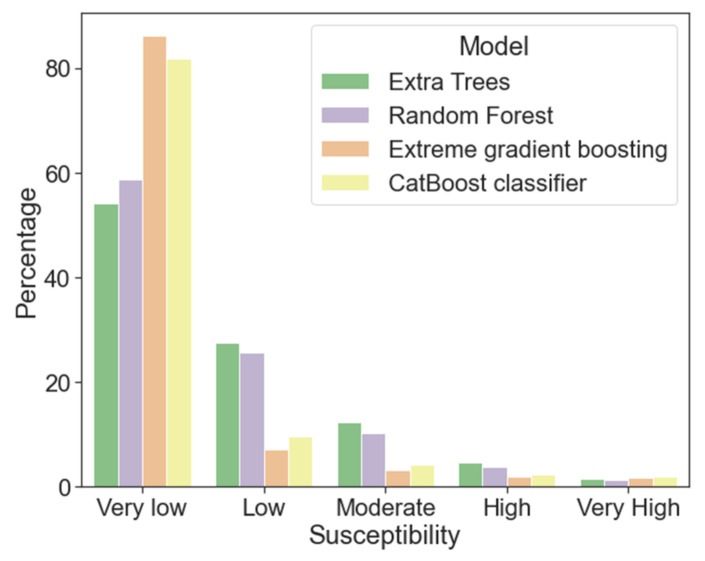
Area percentage of each susceptibility category.

Figure 9 shows the landslide susceptibility map of the entire study area from the four best models. In all cases, it is observed that the areas with the high and high probability of landslides are concentrated in the west of the study area and run through the Sierra Madre del Sur. Furthermore, the areas of greatest susceptibility coincide with those areas where the density of lineaments are high, on lithologies susceptible to landslides. However, exists discrepancies between the predictions of moderate and low classes between bagging models (Figure 9a,b) and boosting models (Figure 9c,d). These differences are based on an overestimation of the very low category in the boosting models. Figure 10 shows the percentage representation of each type of probability to susceptibility developed from the probabilistic prediction of the four best trained models. The classes are ordered from least probable to most probable, finding a range between 56.6% to 87.63% of the study area in zones of very low susceptibility and a range between 1.1% to 1.6% in zones of very high susceptibility.

## 4. Discussion

We compare the results obtained in a sample of current studies developed on modelling susceptibility to landslides. This sample is selected to follow a criterion of relevance, based on the topic of the articles, the number of citations, innovativeness of methods or approach and their availability. In these studies, we observed that the performance of the models when predicting landslides oscillates between 0.602 and 0.958. Regarding the number of explanatory variables used, the ranges oscillate between 10 and 20 variables. The number of landslides identified ranges between 79 and 816, and the study area is between 238.7 and 81,250 square kilometers. Regarding performance, it is observed that the research consulted in the bibliography has not obtained better performance than the present study.

Commonly, in the literature, the burden of research falls on the model generation phase and does not pay enough attention to the data. However, this study highlights the importance of understanding the database and the preparation model phases. In our case, using an automatic machine learning framework, the time is significantly reduced in the model generation phase. At the same time, it makes the supervised learning phase more flexible, giving us the option of using the best possible model based on the data from the entrance, study area or triggers. Furthermore, the automatic selection of models makes it possible to identify patterns in algorithms that will work best with the different datasets. These advantages mean that the weight of our research has also fallen on the phase of understanding and preparing the data, allowing highlight the importance of having a detailed and extensive knowledge of the study area, landslides, and a better selection of variables. In this study, it has been revealed the need of having experts in different disciplines who can better understand how landslides work, the regional knowledge (what happened in the study area during 2013) or give the variables a meaning more related to the objective of the study. In this way, we have participated in a multidisciplinary project with researchers from the Universidad Autónoma de Guerrero (Mexico) and the Rey Juan Carlos University (Spain), with a wide knowledge on the region and the processes [52,55,85].

The comparison of the different models (Table 4) shows that the algorithms based on decision trees are the ones which have obtained the best performance. In the same way, the previous studies with the best results have also used algorithms based on decision trees [22,29,33,36], with some exceptions like [13,30]. However, only one identified study had used a model based on extremely randomized trees (extra trees) [25]. Furthermore, being able to know which models achieve the best performance can be useful to carry out advanced techniques of landslide susceptibility mapping based on “blending” or “ensembling” different models [86,87,88].

In the landslides identification, it is important to generate predictions about susceptibility since the greater number of examples results in a greater number of data and, therefore, a more robust model. In the literature review, only one study has identified greater landslides number but reporting no good results due to a selection of inadequate models [21]. Regarding the study area, a study with a larger covered area has been identified in the literature [36], which has good results based on the good selection of the variables and the models used based on decision trees. On the other hand, no satisfying results are observed in the previous studies with smaller study areas and/or a number of identified landslides [37].

In summary, for the comprehensive management of landslide hazards, it is necessary to advance both in modelling susceptibility and identifying landslides in an automated or semi-automated way. Relying on a complete and updated landslide database will play an important role in evaluating and managing landslide risks [89,90].

The inclusion of variables based on Kernel density, instead of only on distance, in addition to the adequate selection and treatment of geological variables, such as lineaments or lithology, has been key to achieving these results. In the same way, studies that have included variables based on density have obtained good results [13,33,34,36,39]. The results obtained could be improved by using the density of the lineaments instead of the proximity to them, as is the case of [13,30], the only two articles included the lineaments as an explanatory variable.

Finally, in-depth knowledge of the landslides triggering processes and the regional knowledge is essential to obtain good results. In this way, it is necessary to improve data sources, for example, precipitation, to capture the triggering processes of landslides. Currently, multi-source frameworks based on multi-satellite, atmospheric reanalysis and gauge precipitation products are being developed to simultaneously correct precipitation occurrence and intensity producing daily precipitation products [91].

## 5. Conclusions

Methods based on machine learning or deep learning in geosciences have been widely used in recent times. Furthermore, the increasing number of natural disasters are causing that more and more research groups combine these two research lines. However, due to the importance of publishing a novel method instead of focusing on measuring the danger to landslides, sometimes the analysis of the susceptibility results is not addressed enough. This study presents a methodology based on the in-depth knowledge and analysis of the causes of landslides and the variables used to predict areas susceptible to landslides that have allowed us obtained good results. Using for modelling an AutoML framework, comparing the performance of 16 machine learning algorithms, the best model obtained (extra trees classifier) reached an AUC of 0.983 and a kappa of 0.954. In this study is observed that models based on decision trees get better results with less time consumed in their adjustment.

Moreover, having large inventories of landslides help models to generalise better and using variables based on kernel densities instead of distances improves the prediction of the models. Our work highlights the importance of understanding the database and processing phases in data science projects. In our case, having a team of multidisciplinary experts in the field has allowed us to have in-depth knowledge of the different dimensions related to landslide phenomena and regional characteristics. In sum, the present susceptibility measurement methodology has proved useful for managing and evaluating the landslides susceptibility in a scenario in which the recurrence of extreme phenomena is increasing. For future works, we will advance in semi-automatic and automatic landslide detection, improve susceptibility mapping through advanced techniques such as “blending” or “ensembling”, and start work in landslide vulnerability assessment.

## Figures and Tables

**Table 1:** Explanatory variables used in the study.

Type	Variable	Source	Resolution (m)
Topography	Slope	SRTM	30
	Aspect	SRTM	30
	Standard curvature	SRTM	30
	Distance to drainage network	SRTM	30
	Density of drainage network	SRTM	30
Climatic	Average annual precipitation	Daymet	1000
Geology	Lithology	INEGI geological vector data	1:250,000
	Distance to lineaments	INEGI geological vector data	30
	Lineament density	INEGI geological vector data	30
Anthropologic	Distance to road infrastructure	INEGI roads vector data	30
	Density of road infrastructure	INEGI roads vector data	30
Vegetation	NDVI	Landsat 8	30
	Land cover	Copernicus Global Land Service	100

**Table 2:** Aspect categorization.

Degree	Cardinal Points	Category
0°–45°	North-East	1
45°–90°	East-North	2
90°–135°	East-South	3
135°–180°	South-East	4
180°–225°	South-West	5
225°–270°	West-South	6
270°–315°	West-North	7
315°–360°	North-West	8
<0°	Flat	9

**Table 3:** Lithotechnical group categorization.

Category	Lithotechnicall Groups
1	Sedimentary materials (sands, silts and/or conglomerates)
2	Volcanic-sedimentary igneous materials (tuffs, breaches, volcanoclastic)
3	Volcanic igneous materials (andesites, basalts, dacites)
4	Plutonic igneous materials (granites, granodiorites, syenites)
5	Metamorphic materials (quartzites)
6	Sedimentary materials (limestones)
7	Sedimentary materials (gypsum and carbonates)

**Table 4:** Results of the model comparison carried out in this study on the test data.

Model	Accuracy	AUC	Recall	Prec.	F1	Kappa	TT (s)
Extra Trees Classifier	**0.977**	**0.983**	0.983	**0.973**	**0.978**	**0.954**	0.772
Random Forest Classifier	0.976	0.980	0.985	0.971	**0.978**	0.953	1.397
Extreme Gradient Boosting	0.975	0.979	0.988	0.964	0.976	0.949	1.938
Catboost Classifier	0.972	0.977	**0.989**	0.959	0.974	0.945	10.964
Light Gradient Boosting Machine	0.964	0.967	0.981	0.951	0.966	0.928	0.522
Decision Tree Classifier	0.944	0.949	0.959	0.937	0.947	0.889	0.145
MLP Classifier *	0.937	0.943	0.959	0.923	0.941	0.874	35.988
Gradient Boosting Classifier	0.892	0.902	0.915	0.882	0.898	0.783	2.68
SVM—Radial Kernel *	0.894	0.900	0.935	0.871	0.902	0.787	19.744
K Neighbors Classifier *	0.899	0.898	0.972	0.854	0.909	0.796	0.746
Ada Boost Classifier	0.821	0.830	0.823	0.831	0.827	0.640	0.946
Quadratic Discriminant Analysis *	0.799	0.797	0.858	0.779	0.816	0.594	0.045
Logistic Regression *	0.780	0.792	0.812	0.777	0.794	0.559	2.786
Linear Discriminant Analysis *	0.775	0.788	0.818	0.766	0.791	0.547	0.058
Naive Bayes *	0.748	0.756	0.860	0.715	0.781	0.491	0.031
Ridge Classifier *	0.775		0.818	0.766	0.791	0.547	**0.025**

* Algorithms in which the data set is standardized.
